# Reference values of thirty-one frequently used laboratory markers for 75-year-old males and females

**DOI:** 10.3109/03009734.2011.644873

**Published:** 2012-08

**Authors:** Ingvar Ryden, Lars Lind, Anders Larsson

**Affiliations:** ^1^Department of Medical Sciences, Clinical Chemistry, Uppsala University, Uppsala, Sweden; ^2^Department of Medical Sciences, Internal Medicine, Uppsala University, Uppsala, Sweden

**Keywords:** Clinical chemistry, geriatrics, immunoassays, laboratory tests

## Abstract

**Background:**

We have previously reported reference values for common clinical chemistry tests in healthy 70-year-old males and females. We have now repeated this study 5 years later to establish reference values also at the age of 75. It is important to have adequate reference values for elderly patients as biological markers may change over time, and adequate reference values are essential for correct clinical decisions.

**Methods:**

We have investigated 31 frequently used laboratory markers in 75-year-old males (*n* = 354) and females (*n* = 373) without diabetes. The 2.5 and 97.5 percentiles for these markers were calculated according to the recommendations of the International Federation of Clinical Chemistry.

**Results:**

Reference values are reported for 75-year-old males and females for 31 frequently used laboratory markers.

**Conclusion:**

There were minor differences between reference intervals calculated with and without individuals with cardiovascular diseases. Several of the reference intervals differed from Scandinavian reference intervals based on younger individuals (Nordic Reference Interval Project).

## Introduction

During the last fifty years the number of routine laboratory tests has increased dramatically. Today, many clinical laboratories produce more than one million test results per year, and many of these test results are for elderly patients. Elderly patients are often treated by the health care system, and they seek health care more often than individuals representing the age group 20–50 years (1). While the test results are aimed at elderly patients, the reference intervals provided by the laboratory together with the test results are often based on younger individuals. However, it is important to have at its disposal reference intervals that are appropriate for the patient tested. Thus, there is a need to review reference intervals for elderly patients. Regular updating of reference values is generally recommended as old reference values might be inappropriate for today's population, thus resulting in misinterpretation of test results. First, reference values collected decades ago may be outdated due to changes in the population, such as better nutrition or changed degree of activity. Second, reagents are constantly improved and analytical techniques are developed that may change the test results. Third, many of elderly reference intervals may be based on relatively small populations resulting in less accurate reference intervals. Elderly publications also often lack important information regarding reference population, methods used, traceability, and imprecision. Without such information it is difficult to transfer and use published reference values of other laboratories.

For high-volume tests there are international calibrators that reduce intermanufacturer differences. Also, routine laboratories participate in external quality assurance programs that aim to reduce interlaboratory differences (2-4). Results from these quality assurance programs verify that there are good interlaboratory agreements for high-volume laboratory markers. It should thus be possible to have common reference intervals, independent of the laboratory that performs the test. Increasing international communication between health care providers also increases the need for harmonization of laboratory test results including reference intervals (5). Several groups are presently establishing common reference values (6,7).

Many biomarkers change with age, and reference values based on a population in the age range 20–50 years may thus not be appropriate for 75-year-old patients.

We have previously reported reference values from this study cohort at the age of 70 (8). Since appropriate reference values are essential for quality patient care, we felt an urge to reinvestigate reference values for our most widely used serum analytes at the age of 75. The criteria for defining a ‘healthy' population used to establish reference values vary. If very strict criteria are used in an elderly population the group may become very small and not really representative of the whole population. Many elderly individuals are treated with some type of medication and often have had symptoms of cardiovascular disease (CVD). When we set up the selection criteria for this study we decided to exclude all individuals with diabetes mellitus and then calculate reference values both for the whole group and for the subgroup without a history of CVD to be able to compare them with each other.

## Materials and methods

### Subjects

Eligible were all subjects aged 70 living in the community of Uppsala, Sweden. The subjects were chosen from the register of community living and were invited in a randomized order from the start of the study in April 2001 to the last-included subject in June 2005. The subjects received an invitation letter within 1 month of their 70th birthday in order to standardize for age. Of the 2025 subjects invited, 1016 subjects were investigated giving a participation rate of 50.1%. The study is part of the Prospective Investigation of the Vasculature in Uppsala Seniors (PIVUS) study (9).

These subjects were reinvestigated at age 75. At the reinvestigation, 821 participated and provided blood samples. Blood samples were collected in Vacutainer tubes without additives. After clotting, the samples were centrifugated, and the sera were collected and frozen at –80°C. The study was approved by the Ethics Committee of the University of Uppsala, and the participants gave informed consent prior to blood sampling. The study was performed in accordance with the ethical rules for human experimentation that are stated in the Declaration of Helsinki.

### Baseline investigation

The participants were asked to answer a questionnaire about their medical history, smoking habits, and regular medication. All subjects were investigated in the morning after an overnight fast. No medication or smoking was allowed after midnight. During the investigation, the subjects were supine in a quiet room maintained at a constant temperature. CVD was defined as myocardial infarction, stroke, and heart failure, but not hypertension.

### Instrument

Serum alanine aminotransferase (ALT) (EC 2.6.1.2, reagent: 1655281), albumin (reagent: 8196057), alkaline phosphatase (EC 3.1.3.1, reagent: 1053180), apolipoprotein A1 (reagent: 6801737), apolipoprotein B (reagent: 6801738), bilirubin (reagent: 8383051), calcium (reagent: 1450261), chloride (reagent: 8445207), cholesterol (reagent: 1669829), creatinine (reagent: 8141947), creatine kinase (reagent: 8479396), C-reactive protein (reagent: CRP Vario®, Abbott Laboratories, Abbott Park, IL, USA), direct HDL-cholesterol (reagent: 6801895), γ-glutamyltransferase (GGT) (EC 2.3.2.2, reagent: 8257289), iron (reagent: 1515808), lactate dehydrogenase (LDH) (reagent: 838489), pancreatic lipase (EC 3.1.1.3, reagent: 1668409), magnesium (reagent: 825593), phosphate (reagent: 1513209), potassium (reagent: 8157596), sodium (reagent: 8379034), transferrin (reagent: 6801767), triglycerides (reagent: 1336544), uric acid (reagent: 1943927), and urea (reagent: 8102204) measurements were performed on an Ortho Vitros FS 5.1 (Ortho Clinical Diagnostics, Rochester, NY, USA) and reported using SI units. If not stated otherwise within the brackets, the reagents were all obtained from Ortho Clinical Diagnostics. The assays were performed at the department of Clinical Chemistry, Kalmar, Sweden. The laboratory is accredited by Swedac (Borås, Sweden) according to ISO 17025. The assays are included in the Swedish laboratory external quality assurance program managed by Equalis (Uppsala, Sweden). The total analytical imprecision of the assays is presented in Table I, and the traceability of the methods is presented in Table II. From the test results the following variables were also calculated: ApoB/ApoA1 ratio, LDL-cholesterol (LDL-cholesterol = cholesterol – (HDL-cholesterol + Triglycerides/2.2)), LDL/HDL ratio, total iron binding capacity (TIBC) (TIBC (µmol/L) = 25 × transferrin (g/L)), and transferrin saturation (iron saturation (%) = 100 × iron/TIBC).

**Table I.** Total analytical imprecision at two levels for the Vitros methods used to calculate the reference intervals.

Analyte	Concentration (two levels)	Coefficient of variation (CV)
Alanine aminotransferase (ALT)	0.66 µkat/L	4.9%
	3.15 µkat/L	1.3%
Albumin	23 g/L	2.4%
	47 g/L	1.8%
Alkaline phosphatase	1.6 µkat/L	2.8%
	8.5 µkat/L	3.1%
Apolipoprotein A1	1.08 g/L	2.1%
	1.91 g/L	3.7%
Apolipoprotein B	0.89 g/L	2.1%
	1.84 g/L	1.7%
Bilirubin	26 µmol/L	2.9%
	256 µmol/L	1.1%
Calcium	2.1 mmol/L	1.7%
	3.0 mmol/L	1.5%
Chloride	81 mmol/L	0.9%
	105 mmol/L	0.8%
Cholesterol	3.8 mmol/L	1.0%
	6.3 mmol/L	1.0%
HDL-cholesterol	1.0 mmol/L	2.5%
	1.6 mmol/L	2.2%
Creatinine	76 µmol/L	1.3%
	504 µmol/L	1.2%
Creatine kinase	2.7 µkat/L	3.3%
	15.2 µkat/L	4.1%
C-reactive protein (CRP)	5.5 mg/L	5.0%
	84 mg/L	5.9%
γ-Glutamyltransferase (GGT)	1.2 µkat/L	2.6%
	7.4 µkat/L	1.8%
Glucose	4.2 mmol/L	2.5%
	6.5 mmol/L	2.4%
Iron	19 µmol/L	2.2%
	45 µmol/L	1.5%
Lactate dehydrogenase (LDH)	2.7 µkat/L	2.5%
	9.2 µkat/L	1.5%
Lipase	2.7 µkat/L	2.6%
	10.0 µkat/L	1.0%
Magnesium	0.9 mmol/L	1.7%
	1.8 mmol/L	1.2%
Phosphate	1.1 mmol/L	1.4%
	2.3 mmol/L	0.7%
Potassium	3.1 mmol/L	0.9%
	5.4 mmol/L	0.7%
Sodium	117 mmol/L	1.5%
	143 mmol/L	1.7%
Transferrin	1.3 g/L	2.0%
	6.0 g/L	3.7%
Triglycerides	1.3 mmol/L	0.7%
	2.7 mmol/L	1.1%
Urate	250 µmol/L	1.2%
	630 µmol/L	1.4%
Urea	5.6 mmol/L	2.5%
	20.3 mmol/L	2.2%

**Table II.** Traceability of standardizations of methods used to calculate the reference intervals.

Alanine aminotransferase (ALT)	IFCC reference method
Albumin	NIST SRM 927c
Alkaline phosphatase	IFCC reference method
Apolipoprotein A1	WHO-IFCC RR SP1-01
Apolipoprotein B	WHO-IFCC RR SP3-08
Bilirubin	NIST SRM 916a
Calcium	NIST SRM 915a
Chloride	NIST SRM 919a
Cholesterol	NIST 911b
Creatinine	NIST, SRM914
Creatine kinase	Scandinavian Committee on Enzymes
C-reactive protein (CRP)	IRMM/IFCC CRM 470
Glucose	Isotope dilution mass spectrometry (IDMS)
γ-Glutamyltransferase (GGT)	IFCC reference method
HDL-cholesterol	NIST SRM 911b
Iron	NIST SRM 937
Lactate dehydrogenase (LDH)	Pyruvate-to-lactate total lactate dehydrogenase method
Magnesium	NIST SRM 929
Phosphate	NIST SRM 200
Potassium	NIST SRM 918a
Sodium	NIST SRM 919a
Transferrin	IRMM/IFCC CRM 470
Triglycerides	CDC reference method
Urate	NIST SRM 913a
Urea	NIST SRM 912a

Blood glucose was analyzed in whole blood on a HemoCue instrument (HemoCue®, Ängelholm, Sweden) with reagents from the manufacturer.

### Statistical calculations

Calculations of reference values were performed by bootstrap estimation utilizing RefVal 4.0 (Department of Clinical Chemistry, Rikshospitalet, N-0027 Oslo, Norway). RefVal fulfills the recommendations of the International Federation of Clinical Chemistry on the statistical treatment of reference values (10,11). Comparisons between groups were performed with Mann–Whitney *U* test (Statistica, StatSoft, Tulsa, OK, USA).

## Results

### Description of the study population

Basic characteristics of the total samples are presented in Table III. After the exclusion of individuals with diabetes the study population consisted of 373 females (338 without CVD) and 354 males (274 without CVD).

**Table III.** Basic characteristics for the persons included in the reference material (*n* = 727).

	Females, median (IQR)	Males, median (IQR)
Females/males (number)	373	354
Height (cm)	161 (157–165)	175 (172–180)
Weight (kg)	69 (60–78)	79 (73–88)
Waist circumference (cm)	91 (82–98)	93 (86–102)
BMI (kg/m^2^)	26.3 (23.4–29.4)	26.1 (24.0–28.5)
Waist/hip ratio	0.91 (0.87–0.97)	0.97 (0.92–1.01)
DBP (mmHg)	76 (70–80)	76 (70–84)
SBP (mmHg)	150 (140–164)	146 (134–158)

BMI = body mass index; DBP = diastolic blood pressure; SBP = systolic blood pressure.

### Reference values

The 2.5 and 97.5 percentiles for males and females with and without CVD are presented in Table IV. The corresponding values obtained in the Nordic Reference Interval Project (NORIP) are presented in the Appendix (12,13, (available online at http://www.informahealthcare.com/doi/abs/10.3109/03009734.2011.644873)).

**Table IV.** Calculated upper (97.5 percentile) and lower (2.5 percentile) limits for the reference intervals and 90% confidence intervals (in brackets).

ALT (µkat/L)			
Males, all	0.21 (0.18–0.23)	–	0.75 (0.64–0.85)
Males, without CVD	0.20 (0.18–0.23)	–	0.76 (0.62–0.90)
Females, all	0.20 (0.18–0.20)	–	0.60 (0.51–0.69)
Females, without CVD	0.19 (0.18–0.20)	–	0.60 (0.52–0.67)
Albumin (g/L)			
Males, all	37.2 (36.3–38.1)	–	52.5 (51.7–53.2)
Males, without CVD	37.5 (36.7–38.2)	–	52.1 (51.1–53.2)
Females, all	38.2 (37.7–38.8)	–	51.1 (50.3–52.0)
Females, without CVD	38.2 (37.7–38.8)	–	51.1 (50.3–52.3)
Alkaline phosphatase (µkat/L)			
Males, all	0.86 (0.81–0.91)	–	2.19 (1.99–2.39)
Males, without CVD	0.86 (0.80–0.92)	–	2.09 (1.84–2.33)
Females, all	0.84 (0.78–0.90)	–	2.34 (2.04–2.65)
Females, without CVD	0.86 (0.81–0.91)	–	2.42 (2.11–2.74)
Apolipoprotein A1 (g/L)			
Males, all	1.10 (1.07–1.12)	–	2.22 (2.13–2.31)
Males, without CVD	1.11 (1.08–1.14)	–	2.26 (2.10–2.42)
Females, all	1.33 (1.28–1.38)	–	2.50 (2.30–2.71)
Females, without CVD	1.34 (1.29–1.40)	–	2.52 (2.32–2.72)
Apolipoprotein B (g/L)			
Males, all	0.64 (0.59–0.68)	–	1.61 (1.55–1.67)
Males, without CVD	0.66 (0.61–0.71)	–	1.60 (1.54–1.66)
Females, all	0.74 (0.70–0.77)	–	1.71 (1.66–1.76)
Females, without CVD	0.73 (0.69–0.77)	–	1.70 (1.65–1.76)
Apolipoprotein B/Apolipoprotein A1 ratio			
Males, all	0.4 (0.3–0.4)	–	1.2 (1.1–1.2)
Males, without CVD	0.4 (0.3–0.4)	–	1.1 (1.0–1.2)
Females, all	0.3 (0.3–0.4)	–	1.1 (1.0–1.1)
Females, without CVD	0.3 (0.3–0.4)	–	1.1 (1.0–1.1)
Bilirubin (µmol/L)			
Males, all	1.7 (1.6–1.9)	–	18.8 (16.8–20.8)
Males, without CVD	1.8 (1.6–2.0)	–	18.2 (16.3–20.1)
Females, all	1.7 (1.7–1.7)	–	11.8 (11.1–12.6)
Females, without CVD	1.7 (1.7–1.7)	–	11.6 (10.9–12.3)
Calcium (mmol/L)			
Males, all	2.18 (2.16–2.20)	–	2.55 (2.52–2.59)
Males, without CVD	2.19 (2.18–2.20)	–	2.55 (2.52–2.58)
Females, all	2.22 (2.19–2.24)	–	2.57 (2.54–2.60)
Females, without CVD	2.22 (2.20–2.24)	–	2.57 (2.54–2.60)
Chloride (mmol/L)			
Males, all	102 (101–102)	–	111 (110–112)
Males, without CVD	102 (101–103)	–	111 (110–111)
Females, all	99 (98–101)	–	111 (111–112)
Females, without CVD	99 (98–101)	–	111 (111–112)
Cholesterol (mmol/L)			
Males, all	3.2 (3.0–3.4)	–	7.4 (7.2–7.6)
Males, without CVD	3.5 (3.3–3.8)	–	7.4 (7.2–7.6)
Females, all	3.9 (3.6–4.2)	–	7.9 (7.7–8.2)
Females, without CVD	4.1 (3.8–4.4)	–	8.0 (7.7–8.3)
Creatinine (µmol/L)			
Males, all	62 (61–64)	–	133 (123–143)
Males, without CVD	62 (60–64)	–	123 (111–136)
Females, all	53 (51–54)	–	101 (96–107)
Females, without CVD	53 (51–54)	–	98 (93–104)
Creatine kinase (µkat/L)			
Males, all	0.45 (0.35–0.54)	–	4.90 (4.06–5.77)
Males, without CVD	0.44 (0.32–0.56)	–	5.19 (4.12–6.25)
Females, all	0.44 (0.36–0.52)	–	3.35 (2.91–3.80)
Females, without CVD	0.46 (0.37–0.55)	–	3.45 (2.96–3.94)
C-reactive protein (CRP) (mg/L)			
Males, all	0.32 (0.23–0.42)	–	24.11 (16.02–32.20)
Males, without CVD	0.36 (0.25–0.47)	–	23.50 (17.11–29.89)
Females, all	0.37 (0.29–0.45)	–	15.89 (12.46–19.32)
Females, without CVD	0.37 (0.29–0.44)	–	16.49 (11.89–21.10)
γ-Glutamyltransferase (GGT) (µkat/L)			
Males, all	0.29 (0.28–0.31)	–	2.12 (1.87–2.38)
Males, without CVD	0.29 (0.27–0.32)	–	2.06 (1.79–2.33)
Females, all	0.25 (0.24–0.26)	–	1.77 (1.34–2.20)
Females, without CVD	0.25 (0.24–0.26)	–	1.62 (1.14–2.09)
Glucose (mmol/L)			
Males, all	3.8 (3.7–3.9)	–	6.6 (6.2–7.0)
Males, without CVD	3.8 (3.7–4.0)	–	6.5 (6.1–6.8)
Females, all	3.8 (3.6–4.0)	–	6.2 (6.0–6.3)
Females, without CVD	3.7 (3.6–4.0)	–	6.2 (6.0–6.4)
HDL-cholesterol (mmol/L)			
Males, all	0.77 (0.72–0.82)	–	2.29 (2.10–2.48)
Males, without CVD	0.80 (0.75–0.84)	–	2.35 (2.13–2.58)
Females, all	1.02 (0.98–1.06)	–	2.78 (2.57–3.00)
Females, without CVD	1.03 (0.98–1.09)	–	2.82 (2.60–3.04)
Iron (µmol/L)			
Males, all	8.3 (6.8–9.9)	–	34.3 (32.9–35.6)
Males, without CVD	8.4 (6.3–10.4)	–	34.8 (33.3–36.3)
Females, all	8.8 (8.0–9.7)	–	28.8 (27.5–30.1)
Females, without CVD	8.9 (7.9–9.8)	–	28.7 (27.5–30.0)
Lactate dehydrogenase (LDH) (µkat/L)			
Males, all	1.6 (1.5–1.7)	–	3.3 (3.0–3.6)
Males, without CVD	1.6 (1.5–1.7)	–	3.4 (3.0–3.8)
Females, all	1.6 (1.5–1.7)	–	3.4 (3.3–3.5)
Females, without CVD	1.6 (1.5–1.7)	–	3.3 (3.2–3.4)
LDL-cholesterol (mmol/L)			
Males, all	1.4 (1.2–1.7)	–	5.4 (5.1–5.7)
Males, without CVD	1.8 (1.6–1.9)	–	5.4 (5.0–5.7)
Females, all	1.8 (1.6–2.0)	–	5.4 (5.2–5.6)
Females, without CVD	1.8 (1.6–2.0)	–	5.4 (5.1–5.7)
LDL-cholesterol/HDL-cholesterol ratio			
Males, all	0.9 (0.8–1.1)	–	4.6 (4.3–5.0)
Males, without CVD	1.1 (0.9–1.2)	–	4.6 (4.4–4.9)
Females, all	0.9 (0.8–1.0)	–	4.2 (3.9–4.5)
Females, without CVD	0.9 (0.8–1.0)	–	4.1 (3.8–4.5)
Lipase (µkat/L)			
Males, all	0.60 (0.52–0.68)	–	5.61 (4.88–6.34)
Males, without CVD	0.60 (0.48–0.73)	–	5.35 (4.68–6.03)
Females, all	0.74 (0.61–0.87)	–	5.84 (4.99–6.68)
Females, without CVD	0.70 (0.55–0.85)	–	5.66 (4.79–6.53)
Magnesium (mmol/L)			
Males, all	0.72 (0.70–0.74)	–	0.94 (0.92–0.95)
Males, without CVD	0.71 (0.69–0.74)	–	0.94 (0.93–0.96)
Females, all	0.70 (0.68–0.72)	–	0.93 (0.92–0.94)
Females, without CVD	0.70 (0.67–0.73)	–	0.93 (0.92–0.94)
Phosphate (mmol/L)			
Males, all	0.86 (0.82–0.90)	–	1.38 (1.33–1.42)
Males, without CVD	0.84 (0.77–0.90)	–	1.35 (1.32–1.38)
Females, all	0.98 (0.93–1.02)	–	1.47 (1.44–1.50)
Females, without CVD	0.97 (0.92–1.01)	–	1.47 (1.43–1.51)
Potassium (mmol/L)			
Males, all	3.7 (3.6–3.8)	–	4.9 (4.7–5.0)
Males, without CVD	3.7 (3.6–3.8)	–	4.9 (4.7–5.0)
Females, all	3.7 (3.6–3.8)	–	4.8 (4.7–4.8)
Females, without CVD	3.6 (3.5–3.7)	–	4.8 (4.7–4.8)
Sodium (mmol/L)			
Males, all	137 (137–138)	–	146 (145–147)
Males, without CVD	137 (136–138)	–	145 (145–146)
Females, all	135 (134–137)	–	145 (145–146)
Females, without CVD	135 (134–137)	–	145 (145–146)
TIBC (µmol/L)			
Males, all	44.4 (41.6–47.2)	–	82.2 (76.8–87.6)
Males, without CVD	43.8 (41.0–46.7)	–	76.8 (71.6–82.0)
Females, all	45.3 (42.8–47.5)	–	82.3 (78.7–85.9)
Females, without CVD	45.0 (42.4–47.5)	–	79.3 (76.6–82.0)
Transferrin (g/L)			
Males, all	1.79 (1.70–1.88)	–	3.27 (3.06–3.48)
Males, without CVD	1.77 (1.67–1.87)	–	3.06 (2.85–3.27)
Females, all	1.80 (1.70–1.89)	–	3.27 (3.13–3.42)
Females, without CVD	1.79 (1.68–1.89)	–	3.16 (3.06–3.25)
Transferrin saturation (%)			
Males, all	13 (10–16)	–	57 (54–60)
Males, without CVD	13 (9–18)	–	59 (55–63)
Females, all	13 (12–14)	–	50 (45–55)
Females, without CVD	14 (12–16)	–	51 (46–56)
Triglycerides (mmol/L)			
Males, all	0.64 (0.58–0.69)	–	3.14 (2.63–3.66)
Males, without CVD	0.63 (0.58–0.69)	–	2.92 (2.54–3.30)
Females, all	0.60 (0.51–0.69)	–	3.06 (2.48–3.63)
Females, without CVD	0.59 (0.50–0.67)	–	3.18 (2.57–3.78)
Urate (µmol/L)			
Males, all	221 (201–242)	–	495 (474–515)
Males, without CVD	217 (196–238)	–	484 (455–512)
Females, all	178 (170–187)	–	434 (401–467)
Females, without CVD	178 (168–188)	–	422 (397–446)
Urea (mmol/L)			
Males, all	4.00 (3.73–4.27)	–	11.22 (9.75–12.69)
Males, without CVD	3.92 (3.58–4.26)	–	10.51 (9.08–11.94)
Females, all	3.63 (3.51–3.75)	–	9.60 (9.14–10.07)
Females, without CVD	3.62 (3.50–3.74)	–	9.48 (8.74–10.21)

### Mann–Whitney tests comparing males and females with and without CVD

At a *P* level of 0.01 only urate (*P* = 0.00008) showed significant differences between males with and without CVD. Despite the significant difference between males with and without CVD, the calculated reference interval after exclusion of individuals with CVD were within the 90% confidence interval of the reference interval calculated with individuals including CVD.

At the same significance level, cholesterol (*P* = 0.0006), CRP (*P* < 0.0001), transferrin (*P* = 0.006), and apolipoprotein ratio (*P* = 0.0001) showed significant differences between females with and without CVD. However, the reference intervals for these markers after exclusion of females with CVD were still within the 90% confidence interval of the corresponding reference interval for all females.

## Discussion

The definition of appropriate reference values is an important responsibility for clinical laboratories. The task of creating well defined and accurate reference intervals is often costly and time-consuming compared to validating reference values provided by the assay manufacturer. However, such reference intervals often lack individuals older than 60–65 years of age. Following the recommendations of the International Federation of Clinical Chemistry, we have established reference values based on 75-year-old healthy males and females for a group of common serum analytes. The study population was recruited in the county of Uppsala, Sweden, and we have previously reported normal values for this cohort at the age of 70 (8). As the number of individuals with cardiovascular disease (CVD) increases with age and the prevalence of CVD in elderly populations can be very high, it could be debated whether individuals with CVD should be included or not. Exclusion of a large fraction of individuals when calculating a reference interval is not optimal as it may induce a bias when using the results for comparison with patients. However, CVD is a heterogeneous group, and in most cases a history of CVD should not affect the biomarkers used in this study. We thus decided to include individuals with CVD in the reference material and then calculate reference values with and without individuals with CVD. Mann–Whitney tests comparing the groups with and without CVD showed that there were few significant differences between the two groups. For males only urate showed a significant difference. Still, the reference interval limits for urate after exclusion of individuals with CVD remained within the 90% confidence interval for all males. For females, only cholesterol, CRP, transferrin, and apolipoprotein ratio showed significant differences between the groups at the *P* = 0.01 level. However, even if there were significant differences between the groups the 90% confidence interval for the reference values were not separated. The reference values with and without the CVD group are presented in Table IV. We chose to divide the reference values according to sex as most laboratory information systems handle sex-specific reference values. For several of the analytes the sex differences are small, and thus the same reference interval could be used for both sexes.

The reference values were compared with the corresponding values obtained in the Nordic Reference Interval Project presented in the Appendix (11,12), (available online at http://www.informahealthcare.com/doi/abs/10.3109/03009734.2011.644873). In the present study the upper reference for ALT in men was significantly lower than the value reported in NORIP but similar to the previous finding in this cohort at 70 years of age (8). The reference intervals for albumin, alkaline phosphatase, creatinine (females), γ-glutamyltransferase, triglycerides, urate, and urea were slightly higher, while the reference intervals for bilirubin, cholesterol, and iron were slightly lower in comparison with NORIP. Thus, several of the reference values differed in comparison with the NORIP reference intervals.

In conclusion, common reference values are important to facilitate collaboration and reduce errors caused by erroneous interpretation of test results. Several reference values in the literature are old. Considering the changes in society and the health care system, including the laboratory tests used, data that are older than 15–25 years may not be representative for today. In our opinion, reference values should be re-evaluated regularly to ensure that they still are appropriate for the patients of today. Sex- and age-specific reference values should be used to interpret results of laboratory tests.

## Supplementary material available online at

http://www.informahealthcare.com/doi/abs/10.3109/03009734.2011.644873.
