# Supplementary material for: Reference values of thirty-one frequently used laboratory markers for 75-year-old males and females
Source: Ups J Med Sci. 2012 Aug;117(3):264–72. doi: 10.3109/03009734.2011.644873 (PMC3410285; doi:10.3109/03009734.2011.644873)
Supplement: Supplementary file 1 [file UPS-0300-9734-117-264_suppl.pdf]

Appendix

Table 1: All cases of hypoglycemic hemiparesis

No of cases	Age	Sex	Type of diabetes	Glucose level	Right hemiparesis	Left hemiparesis	Imaging study	Abnormality	References
2	34	M	1		1	1	ND		Am J Med Sci 1928;175:756
1	17	M	1		1		ND		Deutsch Med Wschr 1928;54:1375
1	55	F	1		1		ND		N Engl J Med 1931;205:1246
1	46	F	1		1		ND		Paris Med. 1951 Sep 8-15;41(33-34):380-5.
1	22	M	1	2.2	1		Angiography	Neg	Arch Intern Med. Nov 1964;114:680-4.
	4	ND	1	ND		4	CT、Angiography	Neg	
4	7	ND	1	ND			CT、Angiography	Neg	Neurology. Jun 1979;29(6):893-6.
	14	ND	1	ND			CT、Angiography	Neg	
1	40	M	1	1.5	1		Angiography	Neg	Br Med J (Clin Res Ed). 1981 Jan 10;282(6258):132-3.
1	74	F	1	1.2	1		ND		Postgrad Med J. 1982 Aug;58(682):501-2.
1	61	F	alcholics	1.7	1		ND		Ann Emerg Med. 1984 Jul;13(7):529-31.
	56	F	insulinoma	ND	1		ND		
3	ND	ND	insulinoma	ND			ND		Postgrad Med J. 1984 Sep;60(707):577-81.
	ND	ND	insulinoma	ND			ND		
1	64	M	2	1.5	1		XeCT	Left internal capsule	Rinsho Shinkei (Clinical Neurology) 1984;24:320-1 (in Japanese)
34	47.3	ND		1.7	31	3	CT、Angiography	Neg	Ann Neurol. Oct 1985;18(4):510-2.

23	43.7	ND		ND	11	12	CT、Angiography	Neg	Ann Neurol. Oct 1985;18(4):510-2.suspected cases
1	ND	ND	insulinoma	1.4	ND		ND		Presse Med. 1985 Oct 12;14(34):1775-8.
	88	M	alcholics	3.1		1	ND		
3	78	F	2	0.8		1	ND		Ann Neurol. 1985 May;17(5):421-30.
	78	F	2	1.4		1	ND		
1	71	F	2	1.8	1		CT	Neg	J Am Geriatr Soc. 1986 Jun;34(6):479-81.
2	64	F	1	3.9	1		CT、Angiography	Neg	Stroke. Sep-Oct 1987;18(5):944-6.
				1.7	1				
2	62	M	1	1.6	1		CT、Angiography	Neg	
				2.2	1				
1	96	F	2	1.3	1		ND		J Am Geriatr Soc. May 1987;35(5):477.
1	33	F	ND	ND	1		MRI	right putamen	Presse Med. Jul 2 1988;17(26):1368.
1	ND	F	ND	ND	ND		ND		Tidsskr Nor Laegeforen. Sep 30 1989;109(27):2773-4.
1	15	F	1	1.7		1	ND		J Emerg Med. May-Jun 1989;7(3):233-6.
1	80	F	2	0.0	ND		ND		J Am Osteopath Assoc. Jun 1990;90(6):539-41.
1	49	M	1	1.2	1		CT	Neg	BMJ. Feb 10 1990;300(6721):369-70.
4	7	F	1	3.5	1		ND		J Pediatr. 1990 Oct;117(4):575-7.
	3	M	1	3.0	1		ND		
	5	M	1	2.1	1		ND		
	6	M	1	2.0	1		ND		
1	40	F	1	1.9	1		ND		Neurologia. Feb 1992;7(2):77-9.

1	59	M	2	2.2	1		CT	Left internal capsule	Eur Neurol. 1993;33(1):80-2.
1	31	F	2	1.2	1		CT、Angiography、SPECT	left hemisphere	J Neurol Neurosurg Psychiatry. 1993 Jun;56(6):700-1.
1	33	M	1	3.2		1	CT	Neg	Heart Lung. Jul-Aug 1995;24(4):330-2.
54	8.4	22M/22F	1	ND	29	25	CT	bilateral internal capsule	Acta Paediatr. May 1998;87(5):542-4.
9	ND	ND	1	ND	1	4	CT、MRI	Neg	Diabetes Care. Sep 1998;21(9):1567-8.
1	6	F	Accident	1.9	1		ND		J Emerg Med. May-Jun 1998;16(3):433-5.
1	81	F	2	1.7		1	CT	Neg	Med Klin (Munich). 1998 Jun 15;93(6):374-7.
1	83	F	2	2.2	1		CT	Neg	
1	74	F	2	1.0		1	ND		East Afr Med J. 1998 Jan;75(1):53-4.
2	69	M	2	1.4	1		CT	Neg	West J Med. 1999 Mar;170(3):170-1.
				1.9		1	CT	Neg	
1	83	M	2	1.2	1		CT	Neg	Eur J Emerg Med. Jun 1999;6(2):157-9.
1	ND	M	70	2.2	1		MRI、Angiography	MCA	Tohoku Nokekkansyogai Konwakai Gakujutsushukai Kirokushu 2000;22:39-45 (in Japanese)
1	12	F	1	2.3	1		CT	Neg	Pediatr Neurol. May 2001;24(5):385-6.
3	74	F	2	2.7	1		CT, MRI, Angiography	Neg	No To Shinkei. Dec 2001;53(12):1135-9.
	72	M	2	1.9	1		CT, Angiography	Neg	
	82	F	2	2.1		1	CT, MRI,	Neg	

							Angiography		
3	18	F	1	1.9	3		CT, Angiography	Neg	J Natl Med Assoc. Nov 2002;94(11):999-1001.
1	6	M	1	2.8		1	MRI, SPECT	ND	Clinical Pediatric Endocrinology. 2003;12(2):134.
2	79	M	2	1.8	1	1	ND		J R Soc Med. Jan 2004;97(1):26-7.
1	58	M	2	0.9		1	MRI	Pons	Diabet Med. Jun 2004;21(6):623-4.
1	ND	M	alcholics	ND			ND		Afr J Med Med Sci. 2004 Dec;33(4):377-80.
1	77	M	2	1.7		1	MRI	splenium of the corpus callosum and corona radiata	Stroke. Mar 2005;36(3):e20-22.
1	24	F	1	2.2		1	MRI	Right internal capsule	Neurology. Jul 12 2005;65(1):175.
1	7	F	1	3.5	1		SPECT	left hemisphere	Syounika Rinsho (Japanese Journal of Pediatrics), 2003;56(6):1079-82 (in Japanese)
1	68	F	2	1.8	1		MRI	bilateral internal capsule	AJNR Am J Neuroradiol. Sep 2006;27(8):1760-2.
1	53	F	insulinoma	1.0	1		MRI		Shinkei Naika(Neurological Medicine) 2006;64:543-546 (in Japanese)
1	78	F	2	2.1	1		MRI	splenium of the corpus callosum and left internal capsule	Cerebrovasc Dis. 2006;22(4):282-3.
1	79	M	2	1.8	1		ND		Aust Fam Physician. 2006 Oct;35(10):805-8.

1	63	M	2	1.3	1		MRI	splenium of the corpus callosum and left internal capsule	Neurologia medico-chirurgica. 2007.10 2007;47(10):486-8.
1	31	M	1	1.9		1	CT,MRI	bilateral cortical layer	Arq Neuropsiquiatr. Mar 2008;66(1):101-3.
1	18	M	1	3.2	1		MRI	Neg	South Med J. Feb 2009;102(2):200-1.
1	55	F	Solitary fibrous pleural tumor	0.2	1		ND		Med Oncol. 2009;26(2):131-5.
2	60	M	ND	ND	1		MRI	splenium of the corpus callosum and bilateral internal capsule	Neurological Surgery. 2009.05 2009;37(5):473-8.
	69	F	2	ND	1		MRI	splenium of the corpus callosum	
1	88	F	2	2.0	1		SPECT	left hemisphere	Tounyoubyou (Journal of the Japan Diabetes Society) 2009;52:410 (in Japanese)
2	62	M	ND	1.3	1		MRI	Left internal capsule	Rinsho Shinkei (Clinical Neurology) 2009;49:445 (in Japanese)
	87	F	2	1.5	1		MRI	Left internal capsule	
1	87	F	2	1.7	1		MRI	Left internal capsule	J Clin Neurosci. 2009 Mar;16(3):426, 483.

1	57	M	2	ND		1	CT,MRI	Neg	Nihon Naibunpitsugakkai zasshi (The Japanese journal of endocrinology) 2010;86:163 (in Japanese)
1	63	M	2	1.3	1		MRI	splenium of the corpus callosum and left internal capsule	Neurol Med Chir (Tokyo). 2010;50(8):651-4.
1	79	F	2	1.9	1		MRI	Left internal capsule	Nosottyu(Japanese Journal of Stroke) 2010;32:401-5 (in Japanese)
1	89	M	2	1.6	1		ND		This case
201			Ave	1.8	126	65			
				Frequency(%)	66.0	34.0		ND: no data	
								M: male	F: female
								CT: computed tomography	MRI: magnetic resonance imaging
								SPECT: Single-photon emission-Computed tomography	

Table 2: Frequency of hypoglycemic hemiparesis

No of hypoglycemia	No of hemiparesis		References
30	3	insulinoma	*Postgrad Med J.* Sep 1984;60(707):577-81.
13	1	insulinoma	*Presse Med.* Oct 12 1985;14(34):1775-78.
125	3	varied causes	*Ann Neurol.* May 1985;17(5):421-30.
168	7		
Frequency (%)	4.2		

Table 3: Detailed information of cases with imaging abnormality

Age	Sex	Hemiparesis side	abnormal side of imaging study	Abnormal finding at	Imaging study	References
64	M	Right	Left	Internal capsule	XeCT	Rinsho Shinkei (Clinical Neurology) 1984;(24):320-1 (in Japanese)
33	F	Right	Right	putamen	MRI	Presse Med. Jul 2 1988;17(26):1368.
59	M	Right	Left	Internal capsule	CT	Eur Neurol. 1993;33(1):80-82.
31	F	Right	Left	hemisphere	CT, Angiography, SPECT	J Neurol Neurosurg Psychiatry. 1993 Jun;56(6):700-1.
ND	ND	ND	Bilateral	Internal capsule	CT	Acta Paediatr. May 1998;87(5):542-44.
6	M	Left		ND	MRI, SPECT	Clinical Pediatric Endocrinology. 2003;12(2):134.
58	M	Left		pons	MRI	Diabet Med. Jun 2004;21(6):623-4.
77	M	Left		splenium of the corpus callosum and corona radiata	MRI	Stroke. Mar 2005;36(3):e20-22.
24	F	Left	Right	Internal capsule	MRI	Neurology. Jul 12 2005;65(1):175.
68	F	Right	Bilateral	Internal capsule	MRI	AJNR Am J Neuroradiol. Sep 2006;27(8):1760-2.
53	F	Right		ND	MRI	Shinkei Naika(Neurological Medicine) 2006;(64):543-546 (in Japanese)

78	F	Right	Left	Internal capsule and splenium of the corpus callosum	MRI	Cerebrovasc Dis. 2006;22(4):282-3.
63	M	Right	Left	Internal capsule and splenium of the corpus callosum	MRI	Neurologia medico-chirurgica. 2007.10 2007;47(10):486-8.
31	M	Left	Right	cortical layer	CT, MRI	Arq Neuropsiquiatr. Mar 2008;66(1):101-3.
60	M	Right	Bilateral	Internal capsule and splenium of the corpus callosum	MRI	Neurological Surgery. 2009.05 2009;37(5):473-8.
69	F	Right		splenium of the corpus callosum	MRI	
88	F	Right	Left	ND	SPECT	Tounyoubyou (Journal of the Japan Diabetes Society) 2009;52:410 (in Japanese)
62	M	Right	Left	Internal capsule	MRI	Rinsho Shinkei (Clinical Neurology) 2009;49:445 (in Japanese)
87	F	Right	Left	Internal capsule	MRI	
87	F	Right	Left	Internal capsule	MRI	J Clin Neurosci. 2009 Mar;16(3):426, 483.
63	M	Right	Left	Internal capsule and splenium of the corpus	MRI	Neurol Med Chir (Tokyo). 2010;50(8):651-4.

				callosum		
79	F	Right	Left	Internal capsule	MRI	Nosottyu(Japanese Journal of Stroke) 2010;32:401-5 (in Japanese)

	Sum	Rate(%)
Internal capsule	13	65.0
splenium of the corpus callosum	6	30.0
